# A Gal-MµS Device to Evaluate Cell Migratory Response to Combined Galvano-Chemotactic Fields

**DOI:** 10.3390/bios7040054

**Published:** 2017-11-21

**Authors:** Shawn Mishra, Maribel Vazquez

**Affiliations:** Department of Biomedical Engineering, City College of New York, New York, NY 10031, USA; smishra000@citymail.cuny.edu

**Keywords:** SDF-1, retina, electric field, microdevice, nervous system

## Abstract

Electric fields have been studied extensively in biomedical engineering (BME) for numerous regenerative therapies. Recent studies have begun to examine the biological effects of electric fields in combination with other environmental cues, such as tissue-engineered extracellular matrices (ECM), chemical gradient profiles, and time-dependent temperature gradients. In the nervous system, cell migration driven by electrical fields, or galvanotaxis, has been most recently studied in transcranial direct stimulation (TCDS), spinal cord repair and tumor treating fields (TTF). The cell migratory response to galvano-combinatory fields, such as magnetic fields, chemical gradients, or heat shock, has only recently been explored. In the visual system, restoration of vision via cellular replacement therapies has been limited by low numbers of motile cells post-transplantation. Here, the combinatory application of electrical fields with other stimuli to direct cells within transplantable biomaterials and/or host tissues has been understudied. In this work, we developed the Gal-MµS device, a novel microfluidics device capable of examining cell migratory behavior in response to single and combinatory stimuli of electrical and chemical fields. The formation of steady-state, chemical concentration gradients and electrical fields within the Gal-MµS were modeled computationally and verified experimentally within devices fabricated via soft lithography. Further, we utilized real-time imaging within the device to capture cell trajectories in response to electric fields and chemical gradients, individually, as well as in combinatory fields of both. Our data demonstrated that neural cells migrated longer distances and with higher velocities in response to combined galvanic and chemical stimuli than to either field individually, implicating cooperative behavior. These results reveal a biological response to galvano-chemotactic fields that is only partially understood, as well as point towards novel migration-targeted treatments to improve cell-based regenerative therapies.

## 1. Introduction

The coordinated movement of groups of cells is critical for many biological processes at nearly all stages of life [1,2]. Physical and chemical guidance cues play critical roles in mediating these cells’ migratory responses. Many external guidance cues have been identified and studied to elucidate their underlying mechanisms in controlling cell migration [2,3]. Mechanisms of cell migration and cellular interfaces with the surrounding microenvironment are not only significant to cell biological function but also critical to cell-based regenerative therapies. The nesrvous system has long been a target of regenerative therapies from transcranial direct stimulation (TCDS) to tumor treating fields (TTFs), with mixed success [4]. In the visual system, transplantation therapies have been used to introduce replacement cells for diseased or degenerated retinal cells, including retinal pigment epithelium, photoreceptors, and ganglion cells [5,6,7,8,9]. However, successful outcomes of these therapies have been limited by the low numbers of cells able to migrate into and integrate with damaged host retina [9,10]. While numerous studies have explored the use of tissue-engineered bioscaffolds, transplantable biomaterials, and cells with highly specific stem cell-like properties, few have explored the use of migratory mechanisms to enable the required integration of motile transplantable cells.

Galvanotaxis, or the directional migration of cells in response to applied electric fields (EFs), has been well-established in wound healing processes and applied in regenerative therapies for the past decade [11,12,13]. Here, damaged cells release their cellular contents, altering the local electrical potential and establishing EFs in the order of 25–200 mV/mm. This provides directional cues that guide the surrounding cells to migrate in the direction of the highest charge [11], typically in the wound center. The focus on galvanotaxis as a means of regeneration has led to a large subset of studies focused on the strength and nature of the electric fields generated during injury. Here, galvanotaxis has been well-studied using epithelial cells in wound healing [14,15], as disruption of the transepithelial potential is one major causes of endogenous electric potentials [16]. In addition, recent galvanotactic studies have been performed in ocular tissues, to measure the cellular regenerative response as a function of applied electric fields, utilizing retinal pigment epithelium [17,18], the lens epithelium [19], or the corneal epithelium [20,21]. An excellent review [22] has recently detailed current developments in the applications of galvanotaxis to numerous physiological processes, individually as well as in combination with other external stimuli.

Chemotaxis, or the migration of cells in response to chemical concentration gradients, is also fundamental to wound healing processes [23], as well as to development [24,25], cancer metastasis [26,27], and immune response [28,29]. Chemotactic mechanisms have been well studied in the nervous system by our group and others [30,31], and have particular significance to current cell-based therapies [32]. In the visual system, the migration of transplanted neural cells is fundamental to synaptic integration within host retina, as transplanted cells must navigate complex host architecture to connect with neuronal targets [10]. The combination of chemotactic fields with galvanotactic fields in neural cell behavior has only recently been explored. Many of these studies, however, highlight the effect of the two fields in opposition to one another [33,34,35]. The pioneering study by Francis Lin et al. superimposed an electric field on top of a CCL19 gradient and measured the migratory behavior of peripheral blood T Cells [33]. However, the two fields were established in such a way that the cell migratory responses to each signal were in opposition to one another. In this setup, it is only possible to determine the relative strength of the two stimuli in controlling the cell behavior. Since the relative orientation of the two cues will determine whether cooperation or competition will occur, studying only opposition leaves out half of the picture. 

The unique cellular scale and design flexibility of the microfluidic systems makes microdevices well-suited for the quantitative study of cell behavior, as well as the investigation of underlying biological mechanisms. In this study, we present a novel microfluidics system, the Gal-MµS, a device that facilitates the study of chemical and galvanic cell stimulation individually or in a combinatory manner. The device enables direct control of the chemical and electrical stimulation of cells, while concurrently facilitating real-time monitoring of cell behavior. Our study used the Gal-MµS to evaluate the migratory responses of neural cells to electric fields and chemical gradients individually, as well as in combinatory fields of both. The results reveal that galvano-chemotactic fields are able to direct the migration of neural cells significantly more than either field individually. These findings indicate a potential cooperative biological mechanism of galvano-chemotaxis that can be explored to develop migration-targeted strategies to improve cell-based regenerative therapies. 

## 2. Materials and Methods

### 2.1. System Design

**Figure 1:**
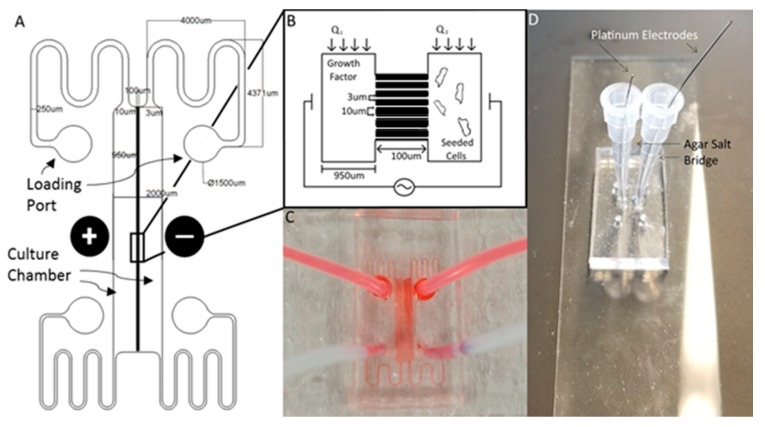
The Gal-MµS. (**A**) Schematic of the design illustrating channel arrays separating two culture chambers. Electrodes are placed on either side of the culture chambers to facilitate controlled application of electric fields. (**B**) Cartoon schematic illustrating Gal-MµS operation, not to scale. Cells are loaded into one culture chamber, while the desired chemical stimulant is loaded into the other. Establishing the flow ratio, Q_1_:Q_2_, provides control of the chemical concentration gradient experienced by cells within the culture chambers. The electrodes positioned on either side of the two culture chambers to enable controlled concurrent electrical stimulation. (**C**) Image of the device (without electrodes) showing fluid flow within culture chambers. (**D**) Image of device demonstrating electrode placement and composition.

**Figure 2:**
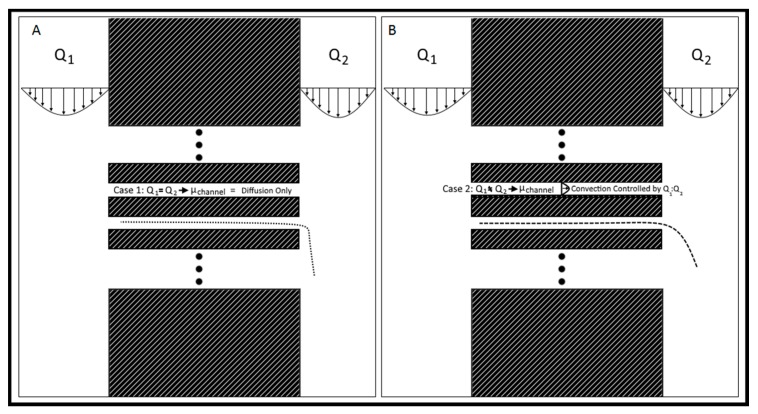
Flow rate controlled chemical gradient. (**A**) Q_1_ = Q_2_, the system is in a state of even flow, resulting in bulk diffusion of chemicals from left to right. The speed of Q_2_ determines the rate of fall-off and degree of accumulation downstream. (**B**) Q_1_ > Q_2_, the system is in a state of uneven flow, resulting in convection-enhanced transport across the channel array from left to right. The rate at which the small molecules cross the channels is controlled by the ratio Q_1_:Q_2_, with larger ratios resulting in faster transport.

The Gal-MµS device was designed to facilitate parallel or uneven flow in two cell culture compartments connected by an array of microchannels (*n* = 760), Figure 1. This system was adapted from a design previously developed by our laboratory to incorporate galvanotaxis in addition to chemotaxis [36]. The two cell culture compartments are 1000 µm-wide by 10^4^ µm-long by 50 µm in height. The culture regions are separated by an array of 100 µm-long channels spaced 10 µm apart, Figure 1A,B. Each channel is 3 µm-wide by 5 µm in height, preventing full bodied cellular migration of neural cells of diameter greater than or equal to 10 µm [37,38], while still facilitating the transport of small molecules from one side to the other. The microchannel array was designed as a barrier to restrict neural cells to their designated seeded culture compartments while enabling transport to generate stable, steady-state chemical concentration gradients across the channel array. The concentration profile, or distribution, of these gradients across the microarray and opposite cell compartments is dependent upon the input flow rates, Q_1_ and Q_2_, Figure 1B. As Q_1_ and Q_2_ are independent of one another, the flow rates can be changed with respect to each other, to provide the desired transport ratios, Q_1_:Q_2_. As seen in Figure 2, controlling this ratio enables the control of the pressure differential across the channel array. The system is in a state of even flow, when Q_1_ = Q_2_ (Figure 2A). The pressure differential between the two sides of the system is equal to zero, and thus the concentration gradient is determined by bulk diffusion. The system is in a state of uneven flow, when Q_1_ ≠ Q_2_ (Figure 2B). In this case, there is a non-zero pressure differential between the two chambers. This results in some pressure-driven flow between the two chambers. Since this pressure differential is dictated by the ratio of Q_1_:Q_2_, we can use that to control the chemical gradient within the culture chambers of the device. Additionally, if the higher flow rate is maintained at less than or equal to 8 dynes, then the impact due to shear stress can be limited. Shear stress was determined for the device previously by our lab [39]. While the flows can be set in counter-flow, all flows used in this study are in a parallel state. Lastly, two columns of agar with an imbedded platinum wire are located on either side of the culture chamber to act as electrodes, Figure 1D.

Chemical environments within the microarray of the so-called H-Channel design have been previously used to study cellular chemotaxis [39], neuron arraying [40], osteocyte communication [36], and cancer metastasis [41]. In the current study, the microarray acts primarily to fluidically isolate the two culture chambers and aid in the creation of a controlled concentration gradient within the cell culture chambers, as had been done previously [40,42]. The channels within the array are small enough to prevent full body cell migration within them but sufficiently large to enable controlled transport of small molecules. As stated above, the controlled transport across the microarray is directly related to the pressure differential between the two culture chambers, as controlled by the relative flow rates—Q_1_ and Q_2_. This enables tight control of the established concentration gradients within the culture chambers.

### 2.2. System Fabrication

**Figure 3:**
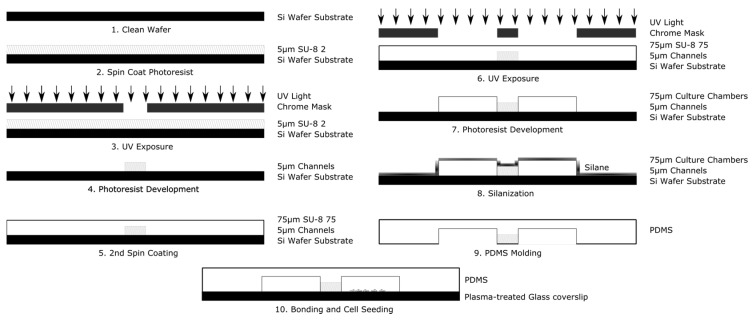
Schematic illustration of the principle steps used in the fabrication of the Gal-MµS device.

The system is fabricated using conventional multistep photolithography to create a master mold and elastomeric molding to stamp this mold into a polymer of polydimethylsiloxane (PDMS), as shown in Figure 3. The design was imprinted onto a 4 in diameter silicon wafer in a 2-step process. The first layer (corresponding to the microarray), composed of the negative photoresist polymer SU-8 2 (MicroChem, Westborough, MA, USA), was first spin-coated at 1000 rpm for 30 s onto the wafer surface using a Laurel WS 650 spin coater (Laurel, North Wale, PA, USA). The wafer was then pre-baked, on a hot plate, at 65 °C for 1 min, followed by an additional 3 min at 95 °C. Exposure was performed using the open channel mask via an automated mask aligner (EVG620, EV Group, Florian/Inn, Austria) with an exposure dose of 100 mJ/cm^2^. The wafer was then post-baked at 65 °C for 1 min followed by an additional minute at 95 °C. The photoresist was developed in PEGMA developer (MicroChem Corp., Westborough, MA, USA) for 5–10 min and rinsed with isopropyl alcohol and deionized water. The wafer was then dried under nitrogen and dehydrated prior to the second step of lithography. The next layer (corresponding to the culture chambers), used photoresist SU-8 2075 (MicroChem, Westborough, MA, USA), was spun on at 4000 rpm for 30 s. The resist was then prebaked for 2 min at 65 °C followed by an additional 7 min at 95 °C. The wafer and second mask were aligned via mask aligner and exposed at 180 mJ/cm^2^. The wafer was then baked for 1 min at 65 °C and 6 min at 95 °C. The wafer was developed in PEGMA developer for ~5–10 min with slight agitation. The wafer was then rinsed with isopropyl alcohol and deionized water, and dried under nitrogen.

To improve contact molding reliability, the finished wafer was then silanized by liquid deposition. The wafer was submerged mixture of methanol, water, and Trichloro(1H,1H,2H,2H-perfluorooctyl) silane mixed at a ratio of 20 mL:1 mL:100 µL, respectively, and gently agitated for 1–2 h. The wafer was rinsed consecutively with isopropyl alcohol and deionized water, and dried with nitrogen. Elastomer molds were constructed with polydimethylsiloxane (PDMS) (Silgard 184, Dow Corning). PDMS prepolymer was mixed at a ratio of 9:1 with its curing agent to a final volume of 30 mL. The ratio of 9:1 prepolymer to PDMS acts to make the final device slightly stiffer than the traditional 10:1 ratio [43]. This allows for easier handling of the device. Additionally, the stiffer polymer holds inserted tubing and pipette tips better, making experimental setup easier for the end user. This mixture was then poured onto the master and degassed for 20 min in a restricted light environment to remove air bubbles. The PDMS was baked until set for 20 min at 75 °C and then demolded. The devices were assembled by ozone plasma, bonding the PDMS casts to cleaned glass coverslips (#1.5, VWR International). The fluidic seal was tested by flowing deionized water or phosphate buffered saline (PBS) at 1 mL/min through both sides of the device via syringe pump (NE-1000, Newera Syringe Pump Systems, Farmingdale, NY, USA). For electric field studies, agar columns with platinum electrodes imbedded were inserted on either side of the culture chambers. The agar powder was first dissolved in Steinberg’s electrolyte solution and then heated to boiling for ~2 min under constant agitation. The agar mixture was cooled for 3–5 min, so as to ease handling. As the agar mixture set, it was injected via syringe into two pipette tips inserted at the edges of the culture two chambers located at the center of the device, so as to come into contact with the media of the culture chambers. Lastly, platinum wires were inserted into the agar-filled pipette tips once the mixture was sufficiently stiff to restrain the electrodes.

### 2.3. Computational Model

A two-dimensional, coupled multiphysics numerical simulation of molecular transport within the Gal-MµS device was performed using both pressure-driven flow and electric fields within the device. These simulations utilized a two-way-coupled finite element model of the device created using COMSOL Multiphysics 5.1 (COMSOL Inc., Burlington, MA, USA). First, the model solves the continuity equations for flow velocity and current density at steady state:

(1) ∇ ·u=0

(2) ∇·i=0

Here ***u*** denotes the velocity (m/s), and ***i*** represents the current-density vector (A/m^2^). The velocity includes two driving forces of pressure and electro-osmotic force given by the velocity equation:

(3) $$u=-\frac{A}{8\mu L}\nabla p+\frac{\epsilon_{w}\zeta }{\mu }\nabla V$$

where *A* is the cross-sectional area of the channel, *µ* is the dynamics viscosity (Pa·s), *L* is the length of the channel (m), *p* is the pressure (Pa), $\epsilon_{w}$ is the fluid’s permittivity (F/m), ζ is the zeta potential (V), and *V* is the electric potential (V). The current density is defined as:

(4) i=−κ∇V

where κ denotes the conductivity (S/m). At the solid walls, the normal velocity component goes to 0:

(5) u·n=0

Flow was modeled as laminar with a mean inflow velocity of 1 × 10^−8^ m/s. This velocity was chosen because it imposes a fluidic shear stress within the culture chambers below the 5 dynes threshold known to affect cell behavior [44]. The electrode voltages were set at ±1 V to achieve an electric field of intensity 100 mV/mm, a biologically representative value [11].

Second, the mass transport within the culture chambers and microarray was modeled via COMSOL’s Transport of Dilute Species package using the velocity and electric potential parameters from the above equations to solve the mass-transport equation for an injected tracer:

(6) $$\frac{\partial c}{\partial t}+\nabla \cdot N=0$$

where *c* is concentration, and ***N*** is the flux vector given by the Nernst-Planck equation [45]:

(7) $$N=-D\nabla c-zu_{m}Fc\nabla V+cu$$

Here D denotes the tracer’s diffusivity (m^2^/s), *c* gives the concentration (mol/m^3^), *z* represents an injected tracer’s partial charge number, *F* is Faraday’s constant (C/mol), and *u_m_* is the mobility of the tracer (mol·m^2^/(J·s)) given by the Nernst-Einstein equation [46]:

(8) $$u_{m}=\frac{D}{R_{g}T}$$

where *R_g_* = 8.314 J/(mol·k) is the gas constant and *T* (K) is the temperature.

An effective diffusivity of 2.14 × 10^−7^ cm^2^/s was used to model the transportation of SDF-1 within the retina, as detailed previously by our group [47]. The effective diffusivity outside of the retinal space was set to 1 × 10^−6^ cm^2^/s, to better approximate the effective coefficient of SDF-1 within water [48]. All physical boundaries of the microsystem were regarded as insulated boundaries of mass transfer, momentum transport, and electron transport. The boundary conditions for the current-density balance are insulating for all boundaries except the electrode surfaces, in which the potential is fixed. 

### 2.4. Cell Culture

Four nervous system cell types and one control cell type were used for this study, as summarized in Table 1. Each cell type was cultured using its own protocol and reagents, as listed below (note that in all experiments, cells were maintained in a bio-incubator at 37 °C and 5% CO_2_, and media was refreshed every 3–4 days until cultured to 95% confluency prior to use). Additionally, cell viability and proliferation for a wide range of nervous system cells within similar scale devices have been shown previously by our group [26,47,49,50], and are expected to be similar here. 

#### 2.4.1. Retinal Progenitor Cells

Multi-passage retinal progenitor cells (RPCs) [56] were cultured in polystyrene culture dishes in Neurobasal medium (NBM; Invitrogen-Gibco, Rockville, MD, USA) containing 2 mM L-glutamine, 100 mg/mL penicillin-streptomycin, 20 ng/mL epidermal growth factor (EGF; Invitrogen-Gibco), and neural supplement (B27 and N2; Invitrogen-Gibco), as per our previous work [47,52]. 

#### 2.4.2. Neonatal Schwann Cells

Primary neonatal Schwann cells (nnSC), obtained from the Thompson lab of RPI [54], were cultured in polystyrene culture dishes in DMEM containing 100 mg/mL penicillin-streptomycin and 10% FBS.

#### 2.4.3. Müller Glial Cells

Multi-passage Muller glia cells (MGC) (ENW0001, Kerafast, Inc., Boston, MA, USA) were cultured in polystyrene culture dishes that had been coated with collagen-1 (A1048301, Thermofisher, Waltham, MA, USA). Cells were cultured in DMEM containing 100 mg/mL penicillin-streptomycin, 4.5 mg/mL L-glutamine, 100 mg/mL penicillin–streptomycin, and 10% FBS. 

#### 2.4.4. Human Umbilical Vein Endothelial Cells

Multi-passage human umbilical vein endothelial cells (HUVECs), obtained from the Barabino lab of CCNY [53], were cultured in polystyrene culture dishes in EGM-2 culture medium (Lonzam, Basel, Swizerland) containing 2% FBS, 0.4% hydrocortisone 0.4% FGF-B, 0.1% VEGF, 0.1% R3-IGF, 0.1% ascorbic acid, 0.1% EGF, 0.1% GA-1000, and 0.1% heparin. 

#### 2.4.5. DAOY

Multi-passage DAOY cells, a medulloblastoma cell line (ATCC# HTB-186) [27,57], were cultured in polystyrene culture dishes in DMEM containing 100 mg/mL penicillin-streptomycin and 10% fetal bovine serum (FBS).

### 2.5. Transwell Migration Assay

Transwell migration assays were performed in 24-well plates (Corning), as described previously by our group [26], using Corning Transwell cell culture insert containing a PET membrane with 8 µm-diameter pores. Cells were starved of FBS (if applicable) 12–18 h prior to the start of the assay. Additionally, for the rMC-1 cells, a collagen I coating (A1048301, ThermoFisher) was seeded onto both sides of the membrane to ensure proper cell adhesion and behavior. 500 µL of serum-free medium containing 100 ng/mL of Stromal Derived Factor-1 (SDF-1) was inserted into the bottom of the assay chamber. Cells were detached and suspended in serum-free medium at a concentration of ~1 × 10^5^ cells/mL, and 300 µL of this solution was inserted into the top of the assay chamber. The cells were then incubated overnight at 37 °C and 5% CO_2_. The number of cells that migrated towards the underside of the membrane were then quantified via fluorescence utilizing CyQuant GR cell proliferation assay kit (C7026, ThermoFisher). The number of motile cells was normalized with respect to the control with serum-free medium, only.

### 2.6. Measurement of Cell Migration in Galvanotactic, Chemotactic, or Combinatory Fields

**Figure 4:**
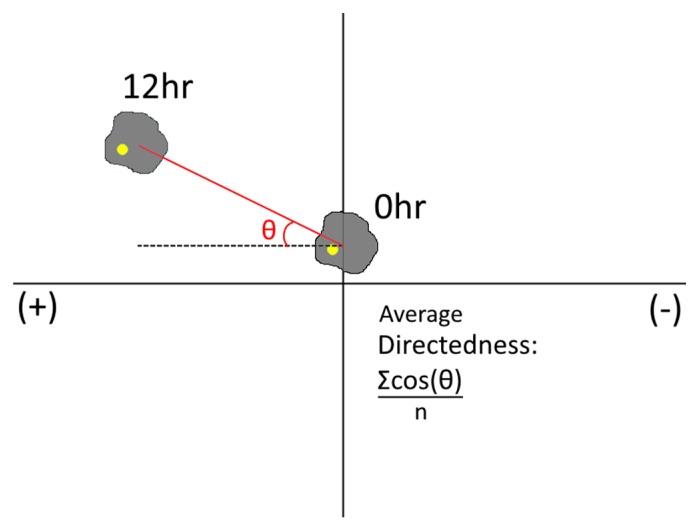
Schematic representation of the angle to calculate directedness.

Each cell type tested was re-suspended in cell culture media at ~1 × 10^5^ cells/mL and injected via syringe pump into one chamber of the Gal-MµS at a rate of 10 µL/min. Concurrently, cell-less media was injected into the remaining chamber at the same rate. This dual injection maintains the pressure balance between the two chambers and allows cells to be evenly seeded into the device. Cells were cultured inside for 2–12 h prior to testing to facilitate cell adhesion. For the rMC-1 cells’ galvanic assay, a col 1 coating was applied prior to cell seeding, after which time cells were subjected to an electric field only of 100 mV/mm for galvanotactic experiments for 12 h, with a constant flow rate of Q = 10 nL/min into both device culture chambers. We note that this flow was necessary to prevent accumulation of cell metabolites and pH changes due to the applied electric field [58]. The electric fields were produced and measured using a NI myDAQ data acquisition device (National Instruments, Austin, TX, USA) and the NI Arbitrary Waveform Generator (National Instruments, Austin, TX, USA). Phase contrast images were gathered every 15 min on a Nikon TE-2000U inverted microscope. For the chemotactic experiments, SDF-1 (100 ng/mL) was flowed into the Gal-MµS at a constant rate of Q = 10 nL/min (into the chamber opposite cells) to establish a constant chemical gradient field. In the experiments using combinatory fields, both the electric field and the SDF-1 gradient were applied, as before, concurrently. In each case, the Gal-MµS was transferred onto the stage of an incubated stage (temperature-controlled, carbon dioxide-controlled, and humidity-controlled) upon a Nikon Eclipse TE2000-U inverted microscope (Morrell Instruments, Melville, NY, USA) for time-lapse imaging. The ImageJ plugin, TrackMate, was used to gather cell trajectories by recording positions of cell centroids over time. At least 45 cells from three independent experiments were tracked for each experimental group. From each of these trajectories, net distance was calculated by subtracting the distance between cell’s initial position and its final position. Additionally, the method of Gruler and Nuccitelli was used to quantify the average cell migration directedness [59]. Migration directedness was used to indicate the degree of alignment between cell migration and an imposed stimulus. Specifically, cell directedness was measured by calculating the cosine of the angle between the imposed electric field lines (as predicted by the computational simulation) and the vector between a cell’s starting position and its final position. As seen in Figure 4, cells that move parallel to the electric field lines have directedness values closer to 1. Cells that move perpendicular to the imposed electric field lines have directedness closer to 0. 

### 2.7. Imaging

Time-lapse migration experiments were performed using a Nikon Eclipse TE2000-U microscope (Morrell Instruments, Melville, NY, USA) fitted with an incubated stage for environmental control of temperature, humidity, and CO_2_, and an automated stage for multi-point image capture. Images were captured every 15 min for 12 h along the center of the device (between the two electrodes). 

### 2.8. Data Analysis

Cell tracking analysis for cell migration was performed using the ImageJ package TrackMate [60]. A Student’s *t*-test and least squares fit were used to measure and analyze the data using MATLAB r2016b (The Mathworks, Inc., Natick, MA, USA). A least squares fit was calculated between the computational concentration profiles and experimentally measured profile. The *t*-test at 95% confidence was performed to determine the disparity between the two concentration gradients, where only *p*-values < 0.05 were considered statistically significant.

## 3. Results and Discussion

### 3.1. Device Design

This study developed the Gal-MµS device to quantitatively stimulate and monitor cells using electric fields, chemical fields, and combinations of both. The Gal-MµS was adapted from a microfluidics-based system previously reported by our laboratory [36], the Macro-micro-nano (Mµn) system. The device consists of a PDMS elastomer bonded to a glass microscope slide, as seen in Figure 1C,D. The device is modeled after a so-called H-ladder design [39] used to generate chemical concentration gradients in a controlled manner. The Gal-MµS is comprised of two distinct culture chambers that are 50 µm-high, 1000 µm-wide, and 10 mm-long. The culture chambers are separated by an array of microchannels that are 5 µm-high, 3 µm-wide, and 100 µm-long, each spaced 10 µm apart. Additionally, agar-agar salt-bridges are imbedded along the center of the device to apply a constant electric field, as well as to prevent electrolyte accumulation inside the medium [61].

The Gal-MµS was designed to achieve fluidic separation between the two cell-seeding regions using the microchannel array. The channel array length was selected as 100 μm to facilitate development of a stable, steady-state concentration gradient between cell-seeded regions. Separation between the two seeding regions is critical to enabling distinct testing conditions of each cell population achieved via even flow or uneven flow, Q_1_ and Q_2_. Setting Q_1_ = Q_2_ even flow is established, in which there is little to no pressure differential between the two culture compartments. This results in molecular diffusion acting as the primary means of mass transport between the two compartments. By altering the ratio of Q_1_:Q_2_ to create uneven flow, a pressure gradient can be established between the two culture compartments. This pressure differential results in convective mass transport towards the lower pressure (i.e., lower flow rate) compartment [37,62]. This pressure-driven diffusional transport enables the controlled stimulation of cells in the adjacent culture compartment.

### 3.2. Computational Model and Experimental Validation

**Figure 5:**
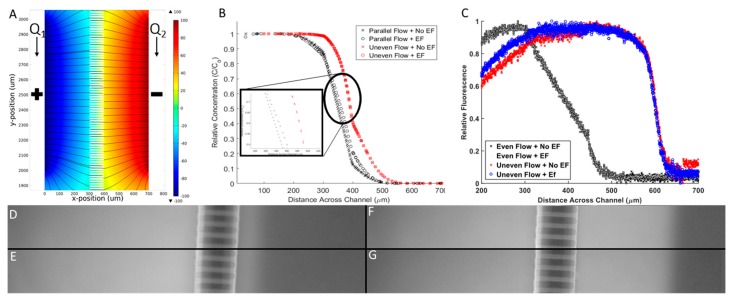
Electrical and chemical microenvironment of Gal-MµS. (**A**) Computationally-derived profiles of electric fields within the Gal-MµS using electric field strength = 100 mV/mm. (**B**) Computationally-derived, steady-state concentration profiles of a model molecule (10 kD dextran) using 4 combinatory fields: even flow (Q_1_ = Q_2_), with and without an applied electric field (100 mV/mm), and uneven flow (Q_1_ > Q_2_), with and without an applied electric field (100 mV/mm). (**C**) Experimental measurement of molecular transport within the device using the 4 combinatory fields. *Note* axes have been modified to better illustrate position of experimental data. Fluorescent images for the 4 stimulation conditions: (**D**) Even flow with no applied electric fields (EF), (**E**) even flow with EF, (**F**) uneven flow with no EF, and (**G**) uneven flow with EF. All images were taken within the region between the two electrodes.

The chemical concentration profiles and electric fields developed within the Gal-MµS were computationally-modeled to quantitatively describe the stimuli that cells experience within the device. The electrodes located in the device center produced a fully-developed and homogenous electric field, as shown in Figure 5A. We note that the EF is only homogeneous closest to the device center, directly between the two electrodes. This restricts the cell monitoring in areas far away from electrodes, as any cells outside of the homogenous electric field would be stimulated in a nonlinear fashion. 

The model also predicted the chemical concentration profile with and without the effect of the electric field for 2 flow conditions. As seen in Figure 5B, the chemical concentration is dramatically reduced from one chamber to the other through the microchannel array. The concentration profile is sigmoidal with a steep linear region near the microchannel array when there is no applied electric field. This result confirms that molecular diffusion is driving transport between the two chambers when equal input flow rates Q_1_, Q_2_ are used, as shown per [40]. When changing from even flow (Q_1_ = Q_2_, black) to uneven flow (Q_1_ > Q_2_, red), a right shift is seen in the concentration profile. Here, the pressure gradient induced convective mass transport to displace larger numbers of molecules across the microarray. In both even flow cases, there is minimal change in the concentration profile when the electric field was applied. This result is a significant and novel aspect of our Gal-MµS. The data confirms that a stable concentration gradient was established across the system and is unaffected by the applied electric field. Numerous systems have applied combinatory electric fields with other stimuli that have resulted in significant EF-altered chemical distribution [22]. Our Gal-MµS provides an environment where both EF and chemical fields are applied in such a way as to separate the biological responses to each.

Results of the computational model were verified via experimental measurements of galvano-chemotactic transport of a model molecule (fluorescent 10 kDa dextran). As seen in Figure 5C, experimental data verified the development of the steady-state concentration profile within the Gal-MµS, with and without electric fields, and with even and uneven parallel flow. Additionally, while the computational model did predict some non-uniformity of the chemical gradient along the outer length of the channel, experiments were performed within the center of the device where the non-uniformity is negligibly small, Figure 5D–G.

The Gal-MµS was designed to provide two culture compartments that are fluidically separated. By maintaining equivalent flow rates through both inlet ports, the pressure differential between the two chambers is negligible. By setting different flow rates we could generate varying steady-state chemical concentration profiles between the two culture chambers, thus controlling the stimuli that the cells were subjected to. Taken together, the computational and experimental results illustrate the predictable profile of small molecules across the array. It has been shown previously that cells stimulated by electric fields can migrate in the direction of one of the stimulating electrodes [13]. Additionally, our lab and others have previously shown that CNS cells migrate along the gradient of a chemoattractant [27,47,48]. The precise control of the concentration profile within the Gal-MµS allows us to quantitatively stimulate cells in a controlled manner, which is ideal for the study of cellular migration.

### 3.3. Galvanotaxis: Electric Field-Induced Migration

**Figure 6:**
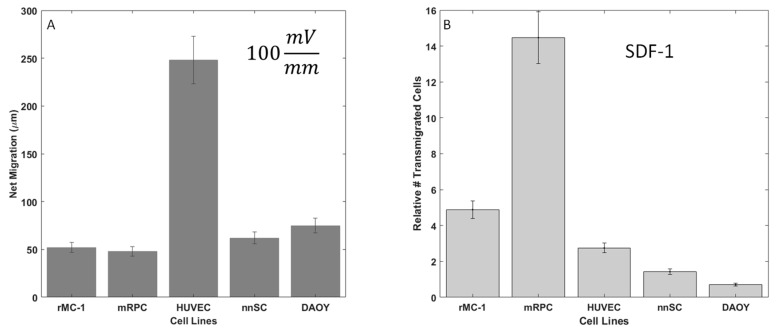
Galvanotactic and chemotactic cell migration of neural cells. (**A**) Average net migration distance of cells in 100 mV/mm electric field. (**B**) Relative number of motile cells in response to Stromal Derived Factor (SDF-1) signaling (100 ng/mL) in transwell migration assays.

The migratory response of 5 cell lines to applied electric fields was measured within the Gal-MµS, as per Table 1: Muller Glia (MGCs), retinal progenitor cells (RPC), Schwann cells (nnSC), Glial Progenitors (DAOY-derived), and Human Umbilical Vein Endothelial Cells (HUVECs). In the absence of electrical stimulation, no measurable movement of cells was observed. Conversely, all cells exhibited migration in response to an applied electric field, as shown in Figure 6A. All neural cells responded with approximately the same net migration distance of 50–80 µm, while HUVECs migrated significantly larger distances of ~250 µm, *p* < 0.01. This agrees with the published literature, as HUVECs are known to migrate strongly in response to electric fields and are often used as the gold standard in galvanotaxis study [12,13]. In addition, HUVEC, DAOY, RPC, and MGC cells migrated towards the cathode, each with high directedness of >0.9. In contrast, nnSC migrated towards the anode with a directedness of 0.88, as listed in Table 2. These directional results agree with what has been previously published for mammalian cells [11,12,13,63,64] and confirm the Gal-MµS’s abilities to evaluate galvanotaxis.

### 3.4. Chemotaxis: Chemokine-Induced Migration

The migratory responses of the 5 cell types to extrinsic growth factor signaling was examined via transwell assay. The migration of all cells to CNTF, EGF, and GDNF revealed that Stromal Derived Factor (SDF-1) resulted in the largest number of motile cells (data not shown). As seen in Figure 6B, significant increases in the relative numbers of motile cells (compared to controls of serum-free media only) were measured in response to the SDF-1 stimulus. This data supports the work reported by our group and others [47,50,56] that illustrates a chemotactic sensitivity of neural cells to SDF-1. As SDF-1 triggered the most dramatic increase in numbers of motile from RPCs, only this cell type was further examined using combinatory galvano-chemotactic fields.

### 3.5. Galvano-Chemotaxis Enhanced Migration

**Figure 7:**
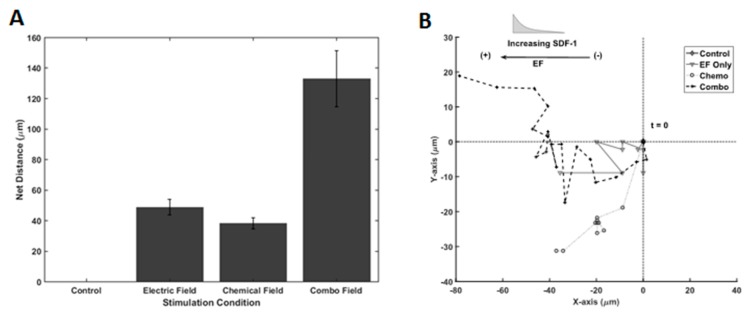
Galvano-, Chemo-, and Galvano-Chemo-Induced migration of RPCs. (**A**) Distance traveled within Gal-MµS device for electrical, chemical, and combinatory stimuli. (**B**) Representative cell trajectories for electrical, chemical, and combinatory stimuli. Trajectories represent 12 h of cell movement within the device.

We next examined the migratory response of RPCs to galvano-chemotactic fields, i.e., the combination of both the SDF-1 gradient and electric field. As shown in Figure 7A, electric field and SDF-1 stimulation, individually, resulted in net migration distances of 48.7 µm ± 5.14 µm and 38.1 µm ± 3.68 µm, respectively, after 12 h of stimulation. Interestingly, RPCs traveled dramatically larger distances in the direction of the electrode in the presence of the combinatory Galvano-chemotactic field, exhibiting a net distance of 133.0 µm ± 18.4 µm. Further, RPCs migrated towards the cathode when stimulated by the electric field and towards increasing gradient when stimulated via SDF-1, which did not change in the combinatory field. Lastly, the trajectories of RPCs, when stimulated by electric fields, SDF-1 gradient fields, and combinatory fields, are shown in Figure 7B. As seen in Table 3, electric field stimulation resulted in a marked increase in directionality compared to chemotactic stimulation, 0.975 vs. 800. This directionality is maintained during combinatory stimulation.

The results demonstrate that the migration of RPCs is dramatically enhanced in response to galvano-chemotactic fields within the Gal-MµS. The average distances traveled were increased nearly 3 times in either individual stimulation case while maintaining high directedness. These results show an increase in net migration that is more than additive as well as an increase in directedness. The changes in these two-different metrics (net migration and directedness) indicate that the electrical and chemical stimuli act cooperatively to produce a migratory response greater than the vector addition of each stimulus. Few studies have focused on the cooperative effects of these two stimuli. Published reports utilize the two fields in opposition to one another so as to study their competing effects [33,34,65,66]. The dominance switching seen in their results suggest that galvanotaxis and chemotaxis pathways share intersecting downstream signaling pathways. Additional studies have proven this overlap [63,67,68]. However, the details mechanism and nature of intersection remain unknown. Further, if the cells’ directionality in each field are considered, then the cooperativity observed in this study agrees with and reinforces the mechanism described in the/literature [34]. Our results suggest the Gal-MµS is well-suited to elucidate the details of this mechanism. Future study of different combinatory stimulation will help evaluate this behavior, mechanistically, and aid the development of migration-targeted, cell-based therapies.

## 4. Conclusions

Cell stimulation via extrinsic signaling of electric fields is a well-established clinical tool used in many physiological systems, including the nervous system. The exciting results of this study suggest that combinatory stimulation with electrical and chemical fields was able to produce cell migratory distance significantly more than that of either field individually. Further study of the cooperative cellular mechanism(s) that facilitate this behavior will greatly aid the development of regenerative therapies in the nervous system.

## Figures and Tables

**Table 1:** Summary of Cells Used.

Cell Name	Type	Comments
MGC	Muller Glia Cell Line	Glial line established from rat retina [51]
RPC	Retinal Progenitor Cell	Primary cells derived from light damage-induced mouse retina [52]
HUVEC	Endothelial Cell Line	Established cell lines used as endothelial cell models [53]
nnSC	Primary Schwann Cells	Primary cells isolated from neonatal mouse dorsal root ganglion [54]
DAOY	Medulloblastoma-derived Cell Line	Transformed glial progenitor from human tumor [55]

**Table 2:** Average Electrotaxis Migration Statistics.

Cell Lines	Muller Glia	HUVEC	RPC	DAOY	Schwann
Average Net Distance (µm)	53.5	246.584	48	60	80
Relative Directedness	0.967 ± 0.012	0.866 ± 0.037	0.999 ± 0.016	0.9 ± 0.022	0.88 ± 0.024
Electrode	Cathode	Cathode	Cathod	Cathode	Anode

**Table 3:** Average Retinal progenitor Cells (RPC) Migration Statistics for Various Stimulation Conditions.

Stimulation Condition	Average Net Distance (µm)	Relative Directedness
Control	0	N/A
Electric Field	48.7 ± 5.14	0.999 ± 0.016
Chemical Field	38.1 ± 3.68	0.800 ± 0.042
Combo	133.0 ± 18.4	0.975 ± 0.026
